# Association between the peripheral neutrophil-to-lymphocyte ratio and metabolic dysfunction-associated steatotic liver disease in patients with type 2 diabetes

**DOI:** 10.3389/fmed.2023.1294425

**Published:** 2023-11-06

**Authors:** Nan Zhu, Yongfeng Song, Chen Zhang, Kai Wang, Junming Han

**Affiliations:** ^1^Department of Endocrinology, Shandong Provincial Hospital Affiliated to Shandong First Medical University, Jinan, Shandong, China; ^2^Shandong Clinical Research Center of Diabetes and Metabolic Diseases, Jinan, Shandong, China; ^3^Shandong Institute of Endocrine and Metabolic Diseases, Jinan, Shandong, China; ^4^Shandong Engineering Laboratory of Prevention and Control for Endocrine and Metabolic Diseases, Jinan, Shandong, China; ^5^Central Hospital Affiliated to Shandong First Medical University, Jinan, China

**Keywords:** metabolic dysfunction-associated steatotic liver disease, type 2 diabetes, neutrophil-to-lymphocyte ratio, logistic regression analysis, risk factor

## Abstract

**Background:**

Metabolic dysfunction-associated steatotic liver disease (MASLD) and type 2 diabetes frequently co-occur, imposing a tremendous medical burden. A convenient and effective MASLD indicator will be beneficial to the early diagnosis of disease. In the clinical laboratory, the neutrophil-to-lymphocyte ratio (NLR) is a readily accessible hematological marker. This study designed to determine the relation between the NLR and MASLD in type 2 diabetes patients.

**Methods:**

Data from 1,151 type 2 diabetes inpatients without infections, malignancy or hematological diseases who were recruited from 2016 through 2022 were analyzed in the retrospective study. The patients were stratified into NLR tertiles (total population: high NLR level > 2.18; middle NLR level: 1.58–2.18; low NLR level < 1.58), with additional subgroup stratification by sex (men: high NLR level > 2.21; middle NLR level: 1.60–2.21; and low NLR level < 1.60; women: high NLR level > 2.12; middle NLR level: 1.53–2.12; and low NLR level < 1.53). After adjusting for confounders (age, sex, weight, Glu, ALT and TG) associated with MASLD, the odds ratio (OR) and the corresponding 95% confidence interval (CI) of the NLR were obtained by using a binary logistic regression analysis to verify the correlation between the NLR and MASLD.

**Results:**

Compared to non-MASLD patients, MASLD patients had higher weight, blood glucose, insulin and C-peptide, worse liver function (higher ALT and GGT), lower HDL (all *p* < 0.05), and lower NLR (*p* < 0.001). The prevalence of MASLD was 43.75% (high NLR level), 55.21% (middle NLR level) and 52.22% (low NLR level) (*p* < 0.05). Compared to those of the high NLR level, the adjusted ORs and 95% CIs of the middle and low NLR levels were 1.624 (95% CI: 1.141–2.311) and 1.456 (95% CI: 1.025–2.068), for all subjects, while they were 1.640 (95% CI: 1.000–2.689) and 1.685 (95% CI: 1.026–2.766), for men.

**Conclusion:**

A low NLR is associated with a greater risk of MASLD.

## 1. Introduction

Recently, a multi-society Delphi consensus statement published in 2023 that proposed the new term: metabolic dysfunction-associated steatotic liver disease (MASLD). MASLD describes liver disease associated with metabolic abnormalities, which is based on hepatic steatosis, and one of the five criteria, BMI ≥25 kg/m^2^ (≥23 kg/m^2^ in Asian) or waist circumference > 94 cm in men, >80 cm in women, or ethnicity adjusted; Fasting serum glucose ≥100 mg/dL (≥5.6 mmol/L) or 2-h post-load glucose level ≥ 140 mg/dL (≥7.8 mmol/L) or HbA1c ≥5.7% or on specific drug treatment; Blood pressure ≥ 130/85 mmHg or specific drug treatment; Plasma triglycerides ≥150 mg/dL (≥1.70 mmol/L) or specific drug treatment; or Plasma HDL cholesterol <40 mg/dL (<1.0 mmol/L) for men and < 50 mg/dL (<1.3 mmol/L) for women or specific drug treatment (1). MASLD is widely recognized as the most prevalent chronic liver disease, which affects around 30% of the global population (2). The prevalence of MASLD in type 2 diabetes patients is approximately 65% (3). Additionally, previous researches have demonstrated that the prevalence of MASLD shows a remarkable sex disparity, with higher risk among men (4). The diagnostic methods of MASLD are liver biopsy, imaging examination and additional tests (5, 6), but these detection methods suffer from certain imperfections, such as higher price, exposure to trauma, significant complications and dependence on the operator. Ultrasound diagnosis sensitivity may be limited in mild steatosis but is considered adequate for moderate–severe steatosis (7, 8). The quantification accuracy of Controlled Attenuation Parameter (CAP) is limited (9, 10). Magnetic resonance (MR) demonstrates higher accuracy in identifying and quantifying intrahepatic fat but is generally more expensive (7, 11). Although the fatty liver index (FLI) and hepatic steatosis index (HSI) exhibit reasonable sensitivity and specificity, their use as diagnostic methods in clinical practice is not recommended at the present time (7, 12, 13). There is still a lack of convenient and useful markers to help people identify MASLD.

MASLD is a systemic metabolic disease with hepatic and systemic inflammation (5, 14, 15). Currently, there are few proven biological indicators associated with MASLD. Levels of alanine aminotransferase (ALT) are usually seen as a simple indictor for assessing the inflammation of liver. However, previous studies demonstrated that normal ALT levels do not guarantee absence of inflammatory damage to liver tissue, and elevated ALT levels do not necessarily indicate steatohepatitis (16, 17). Therefore, we wanted to explore new biomarkers related to MASLD.

The neutrophil-to-lymphocyte ratio (NLR) is a major inflammatory marker that receiving more and more attention globally, and it has the advantage of being inexpensive and easily accessible over other methods. The NLR is a sensitive indicator of the body’s inflammatory status (18, 19), and numerous studies have suggested that the NLR is correlated with the prognosis of lung cancer (20), hepatocellular carcinoma (21) and other tumors (22), the occurrence of myocardial infarction (23) and sepsis (24), and the severity of COVID-19 (18, 25, 26). Furthermore, there is a sex difference in the NLR values among Chinese adults (27, 28). However, it has not yet been substantiated that the association between the NLR and MASLD.

Increasing researches have demonstrated that MASLD is linked with multiple metabolic disorders, including insulin resistance, obesity and abnormal glucose metabolism (29). MASLD and type 2 diabetes frequently occur together (3). Therefore, our study was to explore the relation between peripheral NLR values and MASLD in Chinese type 2 diabetes patients.

## 2. Methods

### 2.1. Study population

We set up a database of type 2 diabetes inpatients at the Shandong Provincial Hospital Affiliated to Shandong First Medical University who were recruited between January 1, 2016, and December 31, 2022. All subjects in this study were type 2 diabetes patients. MASLD was identified by ultrasonographic confirmation of hepatic steatosis, which was based on a multi-society Delphi consensus statement (1). The exclusion criteria are mentioned below: 1. Patients aged below 18 years or above 80 years; 2. Patients with concomitant other liver disease, including chronic viral hepatitis, hepatocellular carcinoma, drug-induced liver injury and autoimmune liver disease; 3. Patients with a history of malignancy or hematological diseases before the study; 4. Patients with acute or chronic infections; 5. Patients with history of severe renal insufficiency; 6. Patients whose clinical and laboratory data are insufficient. Finally, 1,151 patients were eligible for enrollment.

The protocol was approved by the Ethics Committee of the Shandong Provincial Hospital Affiliated to Shandong First Medical University (SWYX: NO. 2023–230) and was designed in accordance with the Helsinki Declaration. No informed consent was needed owing to the retrospective noninterventional study design.

### 2.2. Data collection

The study parameters included age, diastolic blood pressure (DBP), systolic blood pressure (SBP), weight, BMI, and waist circumference (WC). We also collected the laboratory test indicators: Glu (glucose), Ins (insulin), C-Peptide (C-P), glycated hemoglobin (HbA1c), alanine aminotransferase (ALT), aspartate aminotransferase (AST), gamma-glutamyl transpeptidase (GGT), serum creatinine (SCr), serum uric acid (SUA), white blood cell (WBC), red blood cell (RBC), lymphocyte (L), monocyte (M), neutrophil (N), NLR (the NLR was the number of neutrophils divided by the number of lymphocytes), hemoglobin (Hb), platelet (PLT), blood lipid indicators: total cholesterol (TC), triglycerides (TG), high-density lipoprotein-cholesterol (HDL-c), low-density lipoprotein-cholesterol (LDL-c), and admission reasons. Body mass index (BMI) was the weight in kilograms divided by height in meters squared.

### 2.3. Abdominal ultrasonography

Hepatic steatosis was diagnosed by ultrasonic imaging. Ultrasound liver testing was carried out by experienced radiologists. The standard of hepatic steatosis by abdominal ultrasound referred to the standardized criteria established by the Chinese Society of Hepatology, Chinese Medical Association (a 2018 update): diffuse enhancement of near-field echo in the liver, gradual attenuation of far-field echo and intrahepatic ductal structure blurring (30). According to the hepatic steatosis grading proposed by the Chinese Society of Hepatology (31), the attenuation degree of echo attenuation in the posterior field, the intensities of hepatic dotted echoes, and the clarity of intrahepatic portal vein into I (low), II (intermediate), and III (high). The posterior-field echo attenuation in fatty liver patients was further graded: degree I, attenuated by <1/3; degree II, attenuated by 1/3–2/3; and degree III, attenuated by >2/3.

### 2.4. Statistical analysis

Continuous variables were represented as the mean ± standard deviation (SD) if normal distributed, and nonnormally distributed continuous variables were represented by the median (IQR). Categorical variables are presented with frequency distributions (n, %). For the comparison between normal and MASLD groups, we used Kruskal–Wallis analysis or chi-square tests. Participants were classified into NLR tertiles for the total study population (high NLR level > 2.18; middle NLR level: 1.58–2.18; low NLR level < 1.58), for men (high NLR level > 2.21; middle NLR level: 1.60–2.21; low NLR level < 1.60) and for women (high NLR level > 2.12; middle NLR level: 1.53–2.12; low NLR level < 1.53), with the first tertile representing the highest NLR values and the third tertile representing the lowest NLR values. Logistic regression was employed to identify the relation between the risk of MASLD and NLR values. The high NLR level served as the reference category. Both unadjusted and adjusted models were analyzed. Statistical analyses were performed using SPSS version 25.0.

## 3. Results

### 3.1. Baseline characteristics

**Figure 1:**
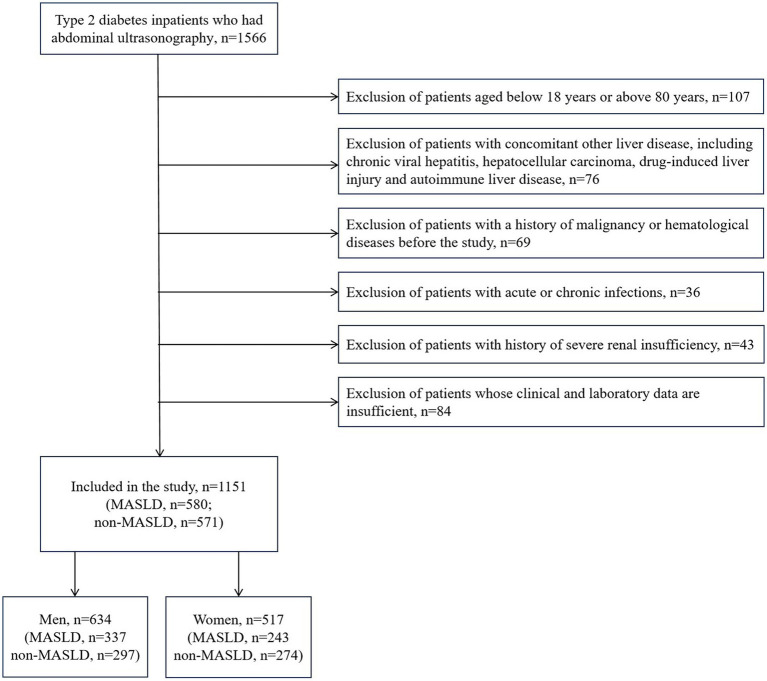
Study flow diagram. MASLD, metabolic dysfunction-associated steatotic liver disease.

The study population contained 1,151 hospitalized type 2 diabetes patients, including 634 men (55.08%) and 517 women (44.92%). See Figure 1 for the study flow diagram. Among the patients, there were 580 MASLD patients and 571 non-MASLD patients. Table 1 lists baseline characteristics. Compared to non-MASLD patients, MASLD patients showed higher weight, blood glucose, insulin and C-peptide levels, worse liver function (higher ALT and GGT), and lower HDL (all *p* < 0.05). Additionally, the NLR in the MASLD group (1.98 ± 0.92) was lower than that in the non-MASLD group (2.26 ± 1.53) (*p* < 0.001). The reasons for hospitalizations in our cohort were type 2 diabetes (82.62%), coronary heart disease (1.48%), cerebral infarction (1.30%), osteoporosis (1.22%), hypertension (0.96%) and others (12.42%). The level of NLR stratification in type 2 diabetes patients is presented in Table 2. The prevalence of MASLD was 43.75% (high NLR level), 55.21% (middle NLR level) and 52.22% (low NLR level) (*p* < 0.05). The NLR in the middle and low NLR levels were significantly higher than those in the high NLR level.

**Table 1:** Clinical characteristics of MASLD and non-MASLD participants.

Characteristics	Diabetic patients		
	non-MASLD	MASLD	P
n	571	580	
age,y	62.05 ± 11.10	56.93 ± 13.15	<0.001
DBP,mmHg	80.27 ± 11.70	83.31 ± 12.08	0.721
SBP,mmHg	133.85 ± 20.25	134.12 ± 18.99	0.204
weight,kg	67.16 ± 11.29	75.88 ± 13.35	0.005
BMI,kg/m^2	24.32 ± 3.30	27.07 ± 3.66	0.106
WC,cm	87.90 ± 10.16	98.54 ± 10.74	0.873
Glu,mmol/L	7.54 (5.91, 9.90)	8.72 (6.93, 11.24)	<0.001
Ins,uU/mL	6.24 (3.49, 11.30)	8.51 (5.03, 14.20)	<0.001
C-P,ng/mL	1.51 ± 1.00	2.15 ± 1.12	<0.001
HbA1c,%	8.39 ± 1.92	8.94 ± 1.96	0.320
ALT,U/L	16.00 (12.00, 23.00)	20.00 (15.00, 29.00)	<0.001
AST,U/L	19.00 (16.00, 23.00)	20.00 (16.00, 25.00)	0.021
GGT,U/L	21.00 (15.00, 29.00)	27.00 (20.00, 38.00)	<0.001
SCr,umol/L	60.72 ± 34.04	58.71 ± 13.49	0.115
SUA,umol/L	290.24 ± 87.51	328.89 ± 94.53	0.037
TC,mmol/L	4.87 ± 1.14	5.28 ± 1.75	0.025
TG,mmol/L	1.18 (0.85, 1.67)	1.64 (1.20, 2.42)	<0.001
HDL-c,mmol/L	1.23 ± 0.31	1.09 ± 0.28	0.013
LDL-c,mmol/L	3.02 ± 0.87	3.33 ± 1.00	0.235
WBC,10^9/L	6.21 ± 1.43	6.52 ± 1.42	0.714
RBC,10^12/L	4.59 ± 0.54	4.80 ± 0.50	0.791
L,10^9/L	1.89 ± 0.60	2.10 ± 0.63	0.754
M,10^9/L	0.44 ± 0.16	0.45 ± 0.15	0.895
N,10^9/L	3.54 (2.88, 4.39)	3.68 (3.03, 4.44)	0.143
NLR	2.26 ± 1.53	1.98 ± 0.92	<0.001
Hb,g/L	137.96 ± 17.47	144.28 ± 15.90	0.304
PLT,10^9/L	230.51 ± 65.18	234.75 ± 59.61	0.033
reasons for admission			<0.001
type 2 diabetes	440 (38.23%)	511 (44.40%)	
coronary heart disease	10 (0.87%)	7 (0.61%)	
cerebral infarction	12 (1.04%)	3 (0.26%)	
osteoporosis	11 (0.96%)	3 (0.26%)	
hypertension	6 (0.52%)	5 (0.43%)	
others	92 (7.99%)	51 (4.43%)	

MASLD, metabolic dysfunction-associated steatotic liver disease; n, number; DBP, diastolic blood pressure; SBP, systolic blood pressure; BMI, body mass index; WC, waist circumference; Glu, glucose; Ins, insulin; C-P, C-Peptide; HbA1c, glycated hemoglobin; ALT, alanine aminotransferase; AST, aspartate aminotransferase; GGT, gamma-glutamyl transpeptidase; SCr, serum creatinine; SUA, serum uric acid; TC, total cholesterol; TG, triglycerides; HDL-c, high-density lipoprotein-cholesterol; LDL-c, low-density lipoprotein-cholesterol; WBC, white blood cell; RBC, red blood cell; L, lymphocyte; M, monocyte; N, neutrophil; NLR, neutrophil/lymphocyte ratio; Hb, hemoglobin; PLT, platelet.

Independent-Samples *T* test or Kruskal-Wallis *H* test.

Normally distributed variables are expressed as the mean ± standard deviation (SD) and nonnormal variables are expressed as the median (IQR).

**Table 2:** Clinical characteristics of patients with type 2 diabetes stratified by NLR tertiles.

Characteristics	Diabetic patients			
	high NLR level	middle NLR level	low NLR level	*P*
*n*	384	384	383	
age,y	61.21 ± 12.04	58.70 ± 12.86	58.49 ± 12.23	0.003
DBP,mmHg	81.78 ± 12.86	82.14 ± 11.53	81.56 ± 11.54	0.781
SBP,mmHg	135.90 ± 20.31	133.72 ± 18.58	132.32 ± 19.77	0.048
weight,kg	71.97 ± 13.62	72.18 ± 12.86	71.50 ± 13.09	0.781
BMI,kg/m^2	25.69 ± 3.88	26.00 ± 3.69	25.70 ± 3.70	0.526
WC,cm	90.50 ± 10.84	99.21 ± 13.51	91.53 ± 8.54	0.004
Glu,mmol/L	8.37 (6.32, 10.93)	8.25 (6.59, 10.51)	7.64 (5.99, 10.32)	0.021
Ins,uU/mL	7.62 (4.49, 13.18)	7.95 (4.55, 12.86)	6.79 (3.64, 12.19)	0.086
C-P,ng/mL	1.90 ± 1.27	1.89 ± 1.01	1.76 ± 1.04	0.199
HbA1c,%	8.63 ± 1.84	8.72 ± 2.01	8.67 ± 2.01	0.848
ALT,U/L	17.00 (12.00, 25.00)	18.00 (13.00, 27.00)	19.00 (14.00, 27.00)	0.016
AST,U/L	19.00 (15.00, 23.00)	19.00 (16.00, 24.00)	20.00 (17.00, 25.00)	<0.001
GGT,U/L	23.00 (16.50, 33.00)	25.00 (17.00, 34.00)	23.00 (17.00, 34.00)	0.200
SCr,umol/L	60.82 ± 30.41	58.68 ± 13.73	59.62 ± 29.81	0.520
SUA,umol/L	304.51 ± 103.17	314.69 ± 89.24	309.80 ± 85.93	0.313
TC,mmol/L	4.86 ± 1.23	5.10 ± 1.40	5.29 ± 1.76	<0.001
TG,mmol/L	1.34 (0.97, 1.86)	1.44 (1.06, 2.16)	1.39 (0.96, 2.06)	0.031
HDL-c,mmol/L	1.14 ± 0.30	1.14 ± 0.29	1.20 ± 0.33	0.007
LDL-c,mmol/L	3.03 ± 0.92	3.20 ± 0.93	3.29 ± 0.99	0.001
WBC,10^9/L	6.79 ± 1.48	6.35 ± 1.28	5.96 ± 1.41	<0.001
RBC,10^12/L	4.61 ± 0.58	4.75 ± 0.51	4.72 ± 0.49	<0.001
L,10^9/L	1.55 ± 0.44	2.02 ± 0.42	2.42 ± 0.66	<0.001
M,10^9/L	0.46 ± 0.16	0.44 ± 0.16	0.43 ± 0.13	0.001
N,10^9/L	4.45 (3.78, 5.47)	3.62 (3.16, 4.22)	2.89 (2.37, 3.46)	<0.001
Hb,g/L	138.15 ± 18.56	142.85 ± 16.22	142.43 ± 15.68	<0.001
PLT,10^9/L	234.53 ± 68.66	232.65 ± 57.60	230.76 ± 60.66	0.718
MASLD	168 (43.75%)	212 (55.21%)	200 (52.22%)	0.004

*n*, number; DBP, diastolic blood pressure; SBP, systolic blood pressure; BMI, body mass index; WC, waist circumference; Glu, glucose; Ins, insulin; C-P, C-Peptide; HbA1c, glycated hemoglobin; ALT, alanine aminotransferase; AST, aspartate aminotransferase; GGT, gamma-glutamyl transpeptidase; SCr, serum creatinine; SUA, serum uric acid; TC, total cholesterol; TG, triglycerides; HDL-c, high-density lipoprotein-cholesterol; LDL-c, low-density lipoprotein-cholesterol; WBC, white blood cell; RBC, red blood cell; L, lymphocyte; M, monocyte; N, neutrophil; Hb, hemoglobin; PLT, platelet; MASLD, metabolic dysfunction-associated steatotic liver disease.

Independent-Samples T test or Kruskal-Wallis H test.

Normally distributed variables are expressed as the mean ± standard deviation (SD), nonnormal variables are expressed as the median (IQR) and categorical variables are presented with frequency distributions (n, %). high NLR level, the highest NLR values (>2.18); middle NLR level, the middle NLR values (1.58–2.18); low NLR level, the lowest NLR values (<1.58).

### 3.2. The relationship between the NLR and the prevalence of MASLD

Table 3 presents the outcomes of the logistic regression. Compared with the high NLR level, the prevalence of MASLD was grossly elevated in the middle and low NLR levels. Compared to the high NLR level, the ORs and 95% CIs of the middle and low NLR levels were 1.585 (95% CI: 1.192–2.107) and 1.405 (95% CI: 1.057–1.867). After adjusting for the clinical variables (age, sex, weight, Glu, ALT, TG) which were demonstrated to be related to MASLD in prior studies (32–34), the NLR remained an independent risk factor for MASLD, and decreased NLR values were related to a higher risk of MASLD. The adjusted ORs and 95% CIs of the middle and low NLR levels vs. the high NLR level were1.624 (95% CI: 1.141–2.311) and 1.456 (95% CI: 1.025–2.068). This suggested that the risk of MASLD in the middle and low NLR levels was 1.624 and 1.456 times higher than that in the high NLR level.

**Table 3:** Unadjusted and adjusted odds ratios of the NLR tertiles for the risk of MASLD in participants.

Tertiles	Unadjusted model		Adjusted model	
	OR (95% CI)	*p*	OR (95% CI)	*p*
high NLR level	/	/	/	/
middle NLR level	1.585 (1.192, 2.107)	0.002	1.624 (1.141, 2.311)	0.007
low NLR level	1.405 (1.057, 1.867)	0.019	1.456 (1.025, 2.068)	0.036

Logistic regression analysis. After adjusting for the clinical variables (age, sex, weight, Glu, ALT, TG).

OR, odds ratio; 95% CI, 95% confidence interval.

high NLR level, the highest NLR values (>2.18); middle NLR Level, the middle NLR values (1.58–2.18); low NLR level, the lowest NLR values (<1.58).

### 3.3. Subgroup analysis by sex

To verify whether sex differences in the correlation between NLR and MASLD, we further investigated a subgroup analysis by sex (Table 4). The NLR values in the separate MASLD groups of men and women were both higher than those in the non-MASLD group (*p* < 0.05). The stratification of the NLR in men and women type 2 diabetes patients is presented in Table 5. The prevalence rate of MASLD showed a significantly increasing trend in men: 45.02% (high NLR level), 56.60% (middle NLR level), and 57.82% (low NLR level), (p < 0.05). No significant increase in women [43.60% (high NLR level), 51.45% (middle NLR level), and 45.93% (low NLR level) (*p* = 0.326)] was found. The outcomes of the logistic regression in men and women are displayed in Table 6. Compared to men with a high NLR, the prevalence of MASLD was significantly elevated in men with a middle or low NLR. Compared with the high NLR level, the ORs and 95% CIs of the middle and low NLR levels in men were 1.593 (95% CI: 1.085–2.338) and 1.674 (95% CI: 1.139–2.460). After adjusting for the clinical variables (age, weight, Glu, ALT, TG), the NLR remained an independent risk factor for MASLD in men. The adjusted ORs and 95% CIs of the middle and low NLR levels vs. those of the high NLR level were 1.640 (95% CI: 1.000–2.689) and 1.685 (95% CI: 1.026–2.766). This suggested that the risk of MASLD in the middle and low NLR levels was 1.640 and 1.685 times higher than that in the high NLR level. The NLR was not an independent risk factor for MASLD in women.

**Table 4:** Clinical characteristics of MASLD and non-MASLD participants according to sex.

Characteristics	Diabetic men (55.08%)			Diabetic women (44.92%)		
	non-MASLD	MASLD	*P*	non-MASLD	MASLD	*P*
*n*	297	337		274	243	
age,y	60.44 ± 11.79	53.07 ± 13.55	0.001	63.79 ± 10.03	62.30 ± 10.45	0.731
DBP,mmHg	81.17 ± 11.80	84.03 ± 11.96	0.842	79.28 ± 11.52	82.29 ± 12.20	0.495
SBP,mmHg	133.66 ± 18.87	132.58 ± 17.86	0.153	134.05 ± 21.71	136.26 ± 20.31	0.940
weight,kg	72.62 ± 9.78	81.62 ± 11.64	0.037	61.20 ± 9.71	67.96 ± 11.38	0.014
BMI,kg/m^2	24.61 ± 3.00	27.21 ± 3.47	0.137	24.03 ± 3.57	26.86 ± 3.92	0.159
WC,cm	88.90 ± 10.82	98.94 ± 10.38	0.475	86.76 ± 9.50	97.71 ± 11.72	0.642
Glu,mmol/L	7.61 (5.99, 10.31)	8.95 (7.20, 11.46)	<0.001	7.43 (5.73, 9.73)	8.43 (6.53, 11.02)	<0.001
Ins,uU/mL	5.81 (3.23, 11.07)	8.63 (4.98, 14.80)	<0.001	7.03 (3.79, 11.44)	8.45 (5.10, 13.57)	0.004
C-P,ng/mL	1.63 ± 1.09	2.28 ± 1.16	0.017	1.35 ± 0.85	1.97 ± 1.04	0.008
HbA1c,%	8.29 ± 1.94	8.87 ± 2.00	0.267	8.50 ± 1.89	9.02 ± 1.91	0.738
ALT,U/L	17.00 (13.00, 25.00)	23.00 (17.00, 33.00)	<0.001	15.00 (11.00, 20.00)	18.00 (13.00, 25.00)	0.001
AST,U/L	19.00 (16.00, 23.00)	20.00 (17.00, 25.50)	0.012	19.00 (16.00, 23.00)	19.00 (16.00, 24.00)	0.599
GGT,U/L	22.00 (17.00, 31.25)	31.00 (23.50, 46.00)	<0.001	18.00 (14.00, 25.00)	21.00 (17.00, 27.00)	<0.001
SCr,umol/L	68.39 ± 41.28	64.46 ± 11.66	0.120	52.41 ± 20.93	50.73 ± 11.67	0.441
SUA,umol/L	317.20 ± 84.70	352.64 ± 97.83	0.047	261.02 ± 81.02	295.78 ± 78.73	0.463
TC,mmol/L	4.64 ± 1.15	5.24 ± 1.86	0.133	5.13 ± 1.07	5.34 ± 1.59	0.018
TG,mmol/L	1.13 (1.66, 0.84)	1.72 (1.22, 2.64)	<0.001	1.26 (0.86, 1.71)	1.48 (1.15, 2.03)	<0.001
HDL-c,mmol/L	1.16 ± 0.29	1.02 ± 0.26	0.028	1.31 ± 0.32	1.20 ± 0.28	0.137
LDL-c,mmol/L	2.87 ± 0.88	3.30 ± 0.94	0.822	3.18 ± 0.83	3.37 ± 1.09	0.012
WBC,10^9/L	6.32 ± 1.35	6.55 ± 1.45	0.292	6.09 ± 1.50	6.48 ± 1.39	0.120
RBC,10^12/L	4.79 ± 0.53	4.98 ± 0.49	0.808	4.37 ± 0.47	4.54 ± 0.39	0.405
L,10^9/L	1.86 ± 0.56	2.09 ± 0.70	0.014	1.93 ± 0.64	2.11 ± 0.52	0.006
M,10^9/L	0.47 ± 0.17	0.47 ± 0.15	0.279	0.41 ± 0.13	0.41 ± 0.14	0.633
N,10^9/L	3.61 (2.96, 4.46)	3.64 (3.03, 4.48)	0.989	3.50 (2.75, 4.32)	3.71 (3.03, 4.41)	0.037
NLR	2.37 ± 1.61	2.03 ± 1.03	0.002	2.15 ± 1.43	1.90 ± 0.73	<0.001
Hb,g/L	145.64 ± 16.06	151.61 ± 13.83	0.300	129.63 ± 14.96	134.13 ± 12.69	0.089
PLT,10^9/L	216.40 ± 56.61	223.19 ± 55.73	0.316	245.80 ± 70.32	250.79 ± 61.20	0.073

MASLD, metabolic dysfunction-associated steatotic liver disease; n, number; DBP, diastolic blood pressure; SBP, systolic blood pressure; BMI, body mass index; WC, waist circumference; Glu, glucose; Ins, insulin; C-P, C-Peptide; HbA1c, glycated hemoglobin; ALT, alanine aminotransferase; AST, aspartate aminotransferase; GGT, gamma-glutamyl transpeptidase; SCr, serum creatinine; SUA, serum uric acid; TC, total cholesterol; TG, triglycerides; HDL-c, high-density lipoprotein-cholesterol; LDL-c, low-density lipoprotein-cholesterol; WBC, white blood cell; RBC, red blood cell; L, lymphocyte; M, monocyte; N, neutrophil; NLR, neutrophil/lymphocyte ratio; Hb, hemoglobin; PLT, platelet.

Independent-Samples T test or Kruskal-Wallis H test.

Normally distributed variables are expressed as the mean ± standard deviation (SD) and nonnormal variables are expressed as the median (IQR).

**Table 5:** Clinical characteristics of NLR stratification in men and women with type 2 diabetes.

Characteristics	Diabetic men (55.08%)				Diabetic women (44.92%)			
	high NLR level	middle NLR level	low NLR level	*p*	high NLR level	middle NLR level	low NLR level	*p*
*n*	211	212	211		172	173	172	
age,y	59.57 ± 13.07	55.43 ± 13.21	54.56 ± 13.04	<0.001	63.52 ± 10.22	63.37 ± 10.83	62.37 ± 9.67	0.524
DBP,mmHg	82.64 ± 12.73	82.97 ± 12.03	82.54 ± 11.12	0.930	80.55 ± 13.20	81.16 ± 11.00	80.47 ± 11.56	0.849
SBP,mmHg	136.32 ± 18.66	132.28 ± 17.34	130.64 ± 18.57	0.006	135.25 ± 22.65	135.60 ± 19.56	134.48 ± 20.96	0.889
weight,kg	77.76 ± 12.58	77.90 ± 11.19	77.52 ± 11.48	0.952	64.12 ± 11.05	65.04 ± 10.73	64.74 ± 11.60	0.773
BMI,kg/m^2	26.17 ± 3.78	26.22 ± 3.36	25.93 ± 3.40	0.729	25.07 ± 3.94	25.77 ± 4.09	25.37 ± 3.96	0.359
WC,cm	93.67 ± 10.05	98.13 ± 13.59	93.00 ± 10.79	0.327	87.33 ± 10.94	98.54 ± 12.72	90.00 ± 5.45	0.025
Glu,mmol/L	8.73 (6.59, 11.47)	8.30 (6.70, 10.57)	7.95 (6.28, 10.92)	0.272	8.03 (6.18, 10.54)	7.98 (6.36, 10.10)	7.54 (5.72, 9.77)	0.118
Ins,uU/mL	7.53 (4.26, 12.76)	8.07 (4.30, 14.00)	6.59 (3.63, 12.34)	0.209	8.04 (5.20, 13.93)	7.76 (4.64, 11.69)	7.09 (3.77, 12.14)	0.284
C-P,ng/mL	2.04 ± 1.40	2.00 ± 1.04	1.93 ± 1.06	0.681	1.73 ± 1.06	1.73 ± 0.94	1.56 ± 1.00	0.252
HbA1c,%	8.52 ± 1.95	8.82 ± 2.03	8.43 ± 1.98	0.266	8.76 ± 1.75	8.67 ± 1.97	8.88 ± 2.02	0.729
ALT,U/L	18.00 (13.00, 28.00)	20.00 (15.00, 30.00)	21.00 (16.00, 29.75)	0.072	15.00 (11.00, 21.00)	16.00 (12.00, 24.50)	17.00 (13.00, 23.50)	0.057
AST,U/L	19.00 (15.00, 24.00)	19.00 (16.00, 24.00)	20.50 (17.00, 25.00)	0.032	18.00 (15.00, 22.00)	19.00 (15.50, 24.00)	20.00 (17.00, 24.00)	0.005
GGT,U/L	25.00 (18.00, 38.00)	28.00 (22.00, 41.00)	28.00 (20.00, 43.75)	0.022	20.00 (15.00, 27.00)	20.00 (14.50, 27.50)	19.00 (15.00, 25.00)	0.676
SCr,umol/L	67.36 ± 32.83	64.37 ± 11.80	67.18 ± 37.53	0.507	52.42 ± 25.01	51.01 ± 12.01	51.43 ± 11.05	0.736
SUA,umol/L	327.59 ± 109.46	337.86 ± 84.52	342.58 ± 84.14	0.243	274.89 ± 86.70	282.80 ± 82.09	274.12 ± 76.30	0.552
TC,mmol/L	4.68 ± 1.19	4.90 ± 1.26	5.29 ± 2.11	<0.001	5.03 ± 1.23	5.39 ± 1.53	5.26 ± 1.23	0.047
TG,mmol/L	1.23 (0.87, 1.86)	1.53 (1.09, 2.38)	1.46 (1.02, 2.39)	0.001	1.40 (1.08, 1.90)	1.33 (0.95, 1.90)	1.32 (0.93, 1.81)	0.497
HDL-c,mmol/L	1.09 ± 0.28	1.06 ± 0.28	1.10 ± 0.30	0.435	1.21 ± 0.31	1.26 ± 0.28	1.31 ± 0.32	0.012
LDL-c,mmol/L	2.92 ± 0.87	3.09 ± 0.86	3.27 ± 1.04	0.001	3.16 ± 0.95	3.36 ± 1.01	3.29 ± 0.92	0.144
WBC,10^9/L	6.83 ± 1.44	6.39 ± 1.35	6.11 ± 1.34	<0.001	6.74 ± 1.51	6.32 ± 1.29	5.76 ± 1.40	<0.001
RBC,10^12/L	4.77 ± 0.57	4.97 ± 0.46	4.93 ± 0.49	<0.001	4.39 ± 0.51	4.45 ± 0.41	4.52 ± 0.40	0.032
L,10^9/L	1.50 ± 0.43	1.99 ± 0.43	2.44 ± 0.69	<0.001	1.61 ± 0.45	2.05 ± 0.43	2.38 ± 0.61	<0.001
M,10^9/L	0.50 ± 0.17	0.47 ± 0.18	0.45 ± 0.13	0.013	0.43 ± 0.14	0.41 ± 0.13	0.40 ± 0.13	0.087
N,10^9/L	4.48 (3.80, 5.53)	3.61 (3.16, 4.34)	2.95 (2.45, 3.54)	<0.001	4.36 (3.76, 5.38)	3.66 (3.13, 4.19)	2.77 (2.22, 3.36)	<0.001
Hb,g/L	144.79 ± 17.08	151.33 ± 12.51	150.29 ± 14.92	<0.001	129.29 ± 16.48	131.74 ± 13.48	134.21 ± 11.57	0.005
PLT,10^9/L	222.31 ± 65.23	218.73 ± 51.18	218.99 ± 51.26	0.766	251.62 ± 69.38	248.08 ± 61.15	244.75 ± 67.93	0.630
MASLD	95 (45.02%)	120 (56.60%)	122 (57.82%)	0.015	75 (43.60%)	89 (51.45%)	79 (45.93%)	0.326

n, number; DBP, diastolic blood pressure; SBP, systolic blood pressure; BMI, body mass index; WC, waist circumference; Glu, glucose; Ins, insulin; C-P, C-Peptide; HbA1c, glycated hemoglobin; ALT, alanine aminotransferase; AST, aspartate aminotransferase; GGT, gamma-glutamyl transpeptidase; SCr, serum creatinine; SUA, serum uric acid; TC, total cholesterol; TG, triglycerides; HDL-c, high-density lipoprotein-cholesterol; LDL-c, low-density lipoprotein-cholesterol; WBC, white blood cell; RBC, red blood cell; L, lymphocyte; M, monocyte; N, neutrophil; Hb, hemoglobin; PLT, platelet; MASLD, metabolic dysfunction-associated steatotic liver disease.

Independent-Samples T test or Kruskal-Wallis H test.

Normally distributed variables are expressed as the mean ± standard deviation (SD), nonnormal variables are expressed as the median (IQR) and categorical variables are presented with frequency distributions (n, %).

Diabetic men: high NLR level, the highest NLR values (>2.21); middle NLR level, the middle NLR values (1.60–2.21); low NLR level, the lowest NLR values (<1.60).

Diabetic women: high NLR level, the highest NLR values (>2.12); middle NLR level, the middle NLR values (1.53–2.12); low NLR level, the lowest NLR values (<1.53).

**Table 6:** Unadjusted and adjusted odds ratios of the NLR tertiles for the risk of MASLD among men and women.

Tertiles	Unadjusted model		Adjusted model	
	OR (95% CI)	*p*	OR (95% CI)	*p*
Men				
high NLR level	/	/	/	/
middle NLR level	1.593 (1.085, 2.338)	0.017	1.640 (1.000, 2.689)	0.050
low NLR level	1.674 (1.139, 2.460)	0.009	1.685 (1.026, 2.766)	0.039
Women				
high NLR level	/	/	/	/
middle NLR level	1.370 (0.897, 2.094)	0.145	1.320 (0.794, 2.194)	0.285
low NLR level	1.099 (0.718, 1.681)	0.665	1.184 (0.714, 1.963)	0.512

Logistic regression analysis. After adjusting for the clinical variables (age, weight, Glu, ALT, TG).

OR, odds ratio; 95% CI, 95% confidence interval.

Men: high NLR level, the highest NLR values (>2.21); middle NLR level, the middle NLR values (1.60–2.21); low NLR level, the lowest NLR values (<1.60).

Women: high NLR level, the highest NLR values (>2.12); middle NLR level, the middle NLR values (1.53–2.12); low NLR level, the lowest NLR values (<1.53).

## 4. Discussion

This retrospective cross-sectional study showed that the NLR was independently, significantly and inversely related to the prevalence of MASLD in type 2 diabetes patients. The study is the first to reveal the relation between the NLR and the risk of MASLD in type 2 diabetes patients.

MASLD is frequently accompanied by increased inflammation (14). Generally, the NLR increases with the initiation and progression of inflammation (19). However, a low NLR was associated with MASLD in the present study. Several possible mechanistic reasons are provided below. First, the low level of neutrophils might be a major factor in oxidative stress in MASLD. Recent animal experimental studies have shown that the inhibition of myeloperoxidase (MPO) can induce oxidative stress (35). Myeloperoxidase is present in primary azurophilic granules of neutrophils (36). Oliviero et al. confirmed a significant positive association between neutrophil proportion and myeloperoxidase in certain types of patients (children with gastroesophageal reflux and asthma-like symptoms) (37). This may suggest that the low level of neutrophils may be accompanied by low MPO expression. Therefore, the low level of neutrophils may play a key role in oxidative stress. Oxidative stress can consume energy and break down DNA, lipids and proteins by impairing mitochondrial function, leading to hepatic inflammation and fibrosis (38). One of the key mechanisms of MASLD is oxidative stress (39), and the adaptive immune reactions induced by oxidative stress play relevant roles in the evolution of MASLD and other diseases toward fibrosis (40). Therefore, a low level of neutrophils has been implicated in the pathogenesis of MASLD. Second, B and T lymphocytes induce the development and progression of MASLD. New evidence suggests that obesity-induced inflammation of visceral adipose tissue can cause glucose intolerance and systemic insulin resistance, and B and T lymphocytes are involved in this process (41, 42). B lymphocytes have a direct effect on the activation of hepatic macrophages and hepatic stellate cells. B lymphocytes stimulate inflammation and fibrosis by multiple interactions with T lymphocytes and hematopoietic stem cells, suggesting that the accumulation of B and T lymphocytes is related to more severe lobular inflammation and enhanced fibrosis (40, 43, 44). Since insulin resistance, inflammation and liver fibrosis are related to the occurrence and development of MASLD (45, 46), we believe that the increase in B and T lymphocytes is related to the onset of MASLD. Third, neutrophils can interact with all kinds of surrounding cell types in the later stage of inflammation and then produce anti-inflammatory lipid mediators, such as lipoxins and resolvins. These lipid mediators inhibit neutrophil activation and recruitment (47). Therefore, we believe that the anti-inflammatory ability of the body may decrease when the NLR is low.

The results demonstrate that in the general population, and particularly in men, a decreased NLR is a risk factor for MASLD, but this association is not significant in women. These effects have been possibly attributed to the protective effects of estrogen: a meta-analysis revealed that the risk of developing MASLD was lower for women than for men (4, 48, 49). Estrogen seems to possess antiadipogenic, antioxidant and antifibrotic properties in the liver. Estrogen increases the expression of miRNA-29a and decreases CCL4 induction in the liver, which may inhibit hepatic steatosis and hepatic fibrosis (50). From experiments with animal models, it is known that estrogen can inhibit astrocyte activation and the formation of fibers (51). Estradiol is an endogenous inhibitor of fibrinolysis that explains sex-related differences in the development of cirrhosis from hepatic fibrosis, and it retards the progression of disease in women (51). Estradiol may also reduce the production of proinflammatory cytokines and prevent macrophage accumulation; therefore, it has anti-inflammatory and antioxidative stress effects (52). Up to this point, we are still not sure of the reasons for this discrepancy, but it is probably due to the smaller sample size of this study, and all patients with type 2 diabetes may conceal some of the evidence.

In addition, this study has several limitations. Firstly, the research was a single-center, cross-sectional study using retrospective data collection. The findings of this study might not be representative of other regions. Therefore, multicenter large-scale prospective studies are required to verify the correlation between the peripheral NLR and MASLD in type 2 diabetes patients. Secondly, the mechanism of the relationship between a decreased NLR and MASLD remains unclear, and we cannot exclude other possible confounders that can result in a decreased NLR. Thirdly, the inflammatory markers, phenotyping of patients and steatosis stratification were not collected for this study, but we will take those variables into account in future studies. Fourthly, this study used abdominal ultrasonography rather than liver biopsy to determine hepatic steatosis. At present, liver ultrasound is still the first-choice imaging diagnostic tool for hepatic steatosis (8, 53). It has a high sensitivity (85%) and specificity (93%) for the diagnosis of moderate-to-severe hepatic steatosis (53). When considering all degrees of steatosis, sensitivity ranges from53.3 to 66.6% and specificity ranges from 77.0 to 93.1% (54).

This study demonstrates that a low NLR is related to the risk of MASLD. This means that a low NLR in type 2 diabetes patients is a potential clinical indicator of MASLD. The NLR is an inexpensive, easily available biomarker that is convenient to promote even in remote regions and is not dependent on the operator, and the NLR can help identify MASLD when people are checking routine blood tests and making interventions. We will investigate the association between the NLR and MASLD in larger population and cohort studies in the near future.

In conclusion, based on our retrospective cross-sectional study, a low NLR may portend increased susceptibility in MASLD patients. The independent association between the NLR and MASLD was proven by a binary logistic regression model. The NLR appears to be a potentially reliable and inexpensive biomarker for the identification of MASLD.

## Data availability statement

The data analyzed in this study is subject to the following licenses/restrictions: The datasets that support the findings of the current study are available from the corresponding author upon reasonable request. Requests to access these datasets should be directed to hanjunming@sdfmu.edu.cn.

## Ethics statement

The studies involving humans were approved by the Ethics Committee of the Shandong Provincial Hospital Affiliated to Shandong First Medical University (SWYX: NO. 2023-230). The studies were conducted in accordance with the local legislation and institutional requirements. The ethics committee/institutional review board waived the requirement of written informed consent for participation from the participants or the participants' legal guardians/next of kin because no informed consent was needed because of the retrospective noninterventional study design.

## Author contributions

NZ: Writing – original draft, Conceptualization, Methodology. YS: Supervision, Writing – review & editing. CZ: Writing – original draft. KW: Writing – original draft. JH: Conceptualization, Writing – review & editing.
